# PD1/PD-L1 blockade in clear cell renal cell carcinoma: mechanistic insights, clinical efficacy, and future perspectives

**DOI:** 10.1186/s12943-024-02059-y

**Published:** 2024-07-16

**Authors:** Zhaoyang Zhu, Yigang Jin, Jing Zhou, Fei Chen, Minjie Chen, Zhaofeng Gao, Lingyu Hu, Jinyan Xuan, Xiaoping Li, Zhengwei Song, Xiao Guo

**Affiliations:** 1 Jiaxing University Master Degree Cultivation Base, Zhejiang Chinese Medical University, Hangzhou, 310000 Zhejiang P.R. China; 2 Department of Urology, The Second Affiliated Hospital of Jiaxing University, Jiaxing, 310000 Zhejiang P.R. China; 3 Department of Surgery, the Second Affiliated Hospital of Jiaxing University, Jiaxing, 310000 Zhejiang P.R. China; 4 Department of General Practice, the Second Affiliated Hospital of Jiaxing University, Jiaxing, 310000 Zhejiang P.R. China

**Keywords:** PD1/PD-L1 inhibitors, ccRCC, Tumor Immune evasion, Combination therapy, Biomarkers

## Abstract

The advent of PD1/PD-L1 inhibitors has significantly transformed the therapeutic landscape for clear cell renal cell carcinoma (ccRCC). This review provides an in-depth analysis of the biological functions and regulatory mechanisms of PD1 and PD-L1 in ccRCC, emphasizing their role in tumor immune evasion. We comprehensively evaluate the clinical efficacy and safety profiles of PD1/PD-L1 inhibitors, such as Nivolumab and Pembrolizumab, through a critical examination of recent clinical trial data. Furthermore, we discuss the challenges posed by resistance mechanisms to these therapies and potential strategies to overcome them. We also explores the synergistic potential of combination therapies, integrating PD1/PD-L1 inhibitors with other immunotherapies, targeted therapies, and conventional modalities such as chemotherapy and radiotherapy. In addition, we examine emerging predictive biomarkers for response to PD1/PD-L1 blockade and biomarkers indicative of resistance, providing a foundation for personalized therapeutic approaches. Finally, we outline future research directions, highlighting the need for novel therapeutic strategies, deeper mechanistic insights, and the development of individualized treatment regimens. Our work summarizes the latest knowledge and progress in this field, aiming to provide a valuable reference for improving clinical efficacy and guiding future research on the application of PD1/PD-L1 inhibitors in ccRCC.

## Intruduction

ccRCC, the predominant subtype of renal cell carcinoma (RCC), comprises about 70–80% of all RCC cases. Globally, the incidence of ccRCC has been rising steadily, with a male-to-female ratio of approximately 2:1. Typically diagnosed between the ages of 50 and 70, ccRCC is associated with several known risk factors, including smoking, hypertension, obesity, chronic kidney disease, and certain genetic conditions such as von Hippel-Lindau (VHL) syndrome [1–3]. Pathologically, ccRCC originates from the epithelial cells of the renal tubules and is characterized by distinct features. The tumor cells exhibit clear cytoplasm due to the accumulation of lipids and glycogen within the cells. ccRCC is often associated with significant angiogenesis, which is closely related to its growth and metastatic potential. ccRCC generally display irregular morphology and highly variable nuclear features, with nuclear grading (Fuhrman grading) being a critical prognostic indicator [4–6]. Clinically, ccRCC presents a wide array of symptoms, often asymptomatic in its early stages. Approximately 25–30% of patients have distant metastases at the time of diagnosis. The most common symptom of ccRCC is hematuria, occurring in about 50–60% of patients, typically presenting as painless hematuria. ccRCC enlargement or invasion into surrounding tissues can lead to persistent or intermittent flank pain. In some cases, a palpable abdominal mass may indicate the tumor has reached a considerable size. Systemic symptoms include weight loss, fever, fatigue, and anemia, reflecting the disease’s extensive impact on the body [7–9]. Due to its resistance to radiotherapy and chemotherapy, surgical intervention, such as nephrectomy, remains the primary treatment for ccRCC. However, approximately 30% of patients experience recurrence or metastasis post-surgery. In recent years, advancements in targeted therapies and immunotherapies, including PD1/PD-L1 inhibitors, have significantly improved treatment options and prognoses for ccRCC patients [10–12].

PD1, an inhibitory receptor, is found on T cells, B cells, and myeloid cells. Its primary ligand, PD-L1, is expressed on the surface of tumor cells and antigen-presenting cells (APCs) [13]. The interaction between PD1 and PD-L1 plays a critical role in maintaining immune homeostasis and preventing autoimmunity by downregulating immune responses. Binding between PD1 and PD-L1 transmits an inhibitory signal that diminishes T cell proliferation, cytokine production, and cytotoxic activity. This mechanism is vital for limiting the immune response during chronic inflammation and minimizing tissue damage [13]. Tumors exploit the PD1/PD-L1 pathway to evade immune surveillance, a process known as immune evasion. By upregulating PD-L1 on their surface, tumor cells can effectively engage PD1 on tumor-infiltrating lymphocytes (TILs), leading to the inhibition of T cell activity. This interaction results in reduced T cell-mediated cytotoxicity and allows tumor cells to evade destruction by the immune system [14].

In ccRCC, PD-L1 expression is often upregulated, which correlates with poor prognosis and increased tumor aggressiveness. The engagement of PD1 by PD-L1 on tumor cells inhibits the anti-tumor immune response, allowing for unchecked tumor growth and progression [15]. Additionally, the tumor microenvironment (TME) in ccRCC is often immunosuppressive, characterized by the presence of regulatory T cells (Tregs), myeloid-derived suppressor cells (MDSCs), and M2-polarized macrophages, all of which contribute to the suppression of effective anti-tumor immunity [16–18]. Understanding the PD1/PD-L1 pathway has led to the development of immune checkpoint inhibitors, which are designed to block this interaction and restore T cell function. By inhibiting PD1 or PD-L1, these therapies aim to enhance the body’s immune response against tumor cells. Clinical trials have demonstrated significant efficacy of PD1/PD-L1 inhibitors in various cancers, including ccRCC, leading to improved survival rates and durable responses in many patients [19–21]. The advent of PD1/PD-L1 inhibitors has revolutionized the treatment landscape for ccRCC, offering new hope for patients with advanced or metastatic disease. These therapies, alone or in combination with other treatments, represent a promising approach to overcoming immune evasion and achieving better clinical outcomes.

With a deeper understanding of the critical role of the PD1/PD-L1 pathway in immune evasion, increasing research efforts are focusing on its specific mechanisms and clinical applications in ccRCC. This review will comprehensively examine the expression and regulatory mechanisms of PD1/PD-L1 in ccRCC, evaluate clinical trial data, and explore combination therapy strategies and related biomarker research advancements. In addition, we discussed possible future research directions in this field. In conclusion, our work aims to provide a valuable reference for improving clinical efficacy and guiding future research on the application of PD1/PD-L1 inhibitors in ccRCC.

## Role and regulation of PD1/PD-L1 pathway in ccRCC

The fundamental mechanisms of the PD1/PD-L1 pathway in ccRCC are largely similar to those observed in other solid tumors. PD1, an inhibitory receptor found on activated T cells, interacts with its ligands PD-L1 and PD-L2, frequently upregulated on tumor cells and various immune cells within the tumor microenvironment. As a result of this interaction, an inhibitory signal is transmitted, leading to reduced T cell proliferation, cytokine production, and cytotoxic activity. This mechanism ultimately facilitates immune evasion by the tumor.

### Expression profile of PD1/PD-L1 in ccRCC

Despite certain commonalities, the expression patterns of PD1 and its ligand PD-L1 in ccRCC are distinct from those in other tumors. For instance, compared to bladder cancer, ccRCC exhibits a higher proportion of PD1 + cells and a relatively lower proportion of PD-L1 + cells within tertiary lymphoid structures (TLS) [6]. Additionally, there is an inconsistency in PD1 and PD-L1 expression between primary and metastatic lesions in ccRCC patients, with higher levels observed in primary tumors [22]. The expression of PD-L1 also varies among different types of ccRCC. For example, the rate of positive PD-L1 expression in VHL-associated hereditary ccRCC is slightly lower than in sporadic ccRCC [23]. Furthermore, studies evaluating PD1 and PD-L1 expression in the peripheral blood of ccRCC patients found that levels of sPD1 and sPD-L1 were lower in cancer patients compared to healthy controls, but higher in metastatic patients compared to non-metastatic ones [24]. At the cellular level, peripheral blood NK cells (PBNK) and tumor-infiltrating NK cells (TINK) in ccRCC patients exhibit distinct PD1/PD-L1 expression patterns. PBNK show a higher frequency of PD1 + cells, whereas TINK display elevated levels of PD1/PD-L1 expression and a more pronounced inhibitory phenotype [25]. Previous studies have developed various risk scoring models to predict PD1/PD-L1 expression. For instance, in one study, 509 ccRCC patients were categorized into two subtypes, C1 and C2, using the expression levels of six HSP genes. The expression levels of HSP90AA1, HSPA8, HSPA1A, and HSPA4L were significantly downregulated in C2 patients. Additionally, C1 patients exhibited markedly lower PD1 expression compared to C2 patients, whereas the expression of PD-L1 was higher in C1 patients [26]. We summarized these studies in Table 1 for a more intuitive presentation.

**Table 1:** Risk score models for predicting PD1/PD-L1 expression

Types of models	Related genes	Expression of PD1/PD-L1	Function	Ref
Prognostic model	DLX4, PLCB2, FIRRE, IL-11, ADH6 and EIF4A1	PD1 and PD-L1 were up-regulated in the high-risk group.	To predict the prognosis of ccRCC patients and the effect of immunotherapy.	[27]
Immune model	SEMA3B, KCNH2, INHA, BPIFA2, FGF19, IL20, GDNF, ANGPTL7, MUC5AC and HLA-DQA1	The expression level of PD1 was lower in the high-risk group.	To predict the overall survival of VHLmut ccRCC patients.	[28]
Lymphangiogenesis prediction model	IL4, CSF2, PROX1 and TEK	PD1 expression was higher in high-risk group	Providing new ideas for the prognosis evaluation and treatment of ccRCC patients.	[29]
Prognostic model	IRF6, TEK, PLCB2, ABCB1, TGFA, COL4A5, PLOD2 and TUBB6	Elevated expression of high-risk groups of PD1.	To predict the clinical prognosis and the efficacy of immunotherapy in ccRCC patients.	[30]
Prognostic model	RARRES2, SOCS3, TNFSF14, XCL1 and GRN	PD-L1 expression increases low-risk group	To predict the prognosis of ccRCC and PD-L1 treatment response	[31]
Immune model	NPR3, MDK, IFNE, NTF4, PTGER1, GAL, FGF23 and CXCL13	The PD1 expression level was higher in the high-risk group.	Stratifying the immune status of patients to facilitate future immunotherapy.	[32]
Prognostic model	CXCL2, SEMA3G, PDGFD and UCN	PD1 expression was higher in high-risk group	To predict the prognosis and treatment effect.	[33]
Prognostic model	MSH3, RAD54L, RAD50, EME1, UNG and NEIL3	The expression of PD1 and PD-L1 was lower in the high-risk group	To predict the OS, PFS and response to immunotherapy in ccRCC patients.	[34]
Prognostic model	LINC00342, AC018752.1, RPL34-AS1, AF117829.1, AC009948.2, SNHG10 and AL133243.3.	The expression of PD1 and PD-L1 increased in the high-risk group	To predict prognosis, immune checkpoint expression and immune cell infiltration.	[35]
Prognostic model	PTPRB, LRP6, ACVR2A, USP2, SLC38A5, NBEA, EMP1, ZNF677, MPZL2, cEVC, ANKS1A, cTMEM72, MDK, SCD5, LIMCH1, FAM13B and RUNX1	The expression of PD1 and PD-L1 increased in the high-risk group.	To predict the prognosis and treatment effect of ccRCC patients.	[36]
Prognostic model	TYROBP, APOC1, CSTA, LY96, LAPTM5, CD300A, ALOX5, C1QA and C1QB	PD1 expression was increased in the high-risk group.	To predict prognosis and benefit from immunotherapy.	[37]
Prognostic model	LINC01270, FIRRE, RP11-37B2.1, RP11-253I19.3, RP11-438L19.1, RP11-504P24.9 and CTB-41I6.1	The high score group had higher expression of PD1/PD-L1	To predict the prognosis of patients with advanced ccRCC.	[38]
Prognostic model	CRABP2, LTB4R, PTGER1 and TEK	PD1 expression was higher in the high-risk group	To predict tumor immune status.	[39]
Prognostic model	AF117829.1, AC108449.2, CHROMR, AL008718.3, COL18A1 − AS1, AL031670.1 and LINC00342	The high risk group had higher expression levels of PD1 and PD-L1	To predict the prognosis and immunotherapy effect of ccRCC patients.	[40]
Prognostic model	ADGRF5, ANO3, GDF7, MYH14, PLCL1, RFTN2, TBC1D1 and ZFP28	PD1 expression was increased in the high-risk group	To predict the prognosis and immunotherapy response of ccRCC patients.	[41]
Prognostic model	ACADM, ACAT1, CPT1B and HACD1	Patients with lower risk scores had higher PD-L1 expression	To predict survival, response to targeted therapy and immunotherapy in ccRCC patients.	[42]
Express prediction model	CSPG4, DNAH11, INADL and TMPRSS13	Carry samples of somatic mutations in PD-L1 positive rate is high	Promising factors predicting positive PD-L1 expression in RCC tumor cells.	[43]
Metastasis prediction and prognostic model	CFB, PPP1R18 and TOM1L1	The high-risk group was more sensitive to anti-PD1 and anti-CTLA-4 immunotherapy, while the low-risk group had a better response to anti-PD-L1 immunotherapy	To predict distant metastasis and response to immunotherapy in ccRCC patients.	[44]
Prognostic model	HSPA1A, HSPA1B, HSPA4L, HSPA8, HSP90AA1 and HSPH1	PD1: C1 < C2 PD-L1: C1 > C2	To predict the clinical prognosis of two subtypes, immune infiltration and drug sensitivity.	[26]

In ccRCC, several factors may be involved in the regulation of PD1/PD-L1 expression. For instance, chemokines such as CCL5 have been implicated in modulating PD1/PD-L1 expression. In ccRCC samples with upregulated CCL5, there is a significant increase in the proportion of CCL5 + tumor-associated macrophages (TAMs) and PD-L1 + CD68 + TAMs, indicative of a typical immunosuppressive tumor immune microenvironment [45]. Additionally, samples with high expression of CCL4 demonstrate increased immune cell infiltration, positively correlated with PD1 expression [46]. Furthermore, factors regulating ferroptosis or cuproptosis may also correlate with PD1/PD-L1 expression. For example, CARS, a potential iron death regulator associated with immune infiltration, exhibits adverse prognosis when highly expressed and positively correlates with PD-L1 expression in ccRCC [47]. The expression of copper apoptosis-related genes CDKN2A and FDX1 correlates positively and negatively with PD1 expression in ccRCC, respectively [48]. Interestingly, in ccRCC, body mass index (BMI) appears to influence PD1/PD-L1 expression, with a negative correlation observed between BMI and PD-L1 expression on tumor cells [49]. Furthermore, BMI shows a negative correlation with the proportions of circulating activated PD1 + CD8 + T cells, CD14 + CD16neg classical monocytes, and Foxp3 + regulatory T cells [50]. Furthermore, certain factors may simultaneously affect the expression of both PD1 and PD-L1. For instance, ARNTL2 is significantly upregulated in ccRCC tissues and cell lines, and its high expression is closely associated with clinical stage progression and unfavorable overall survival rates in ccRCC. High levels of ARNTL2 expression are associated with high infiltration levels of CD8 + T cells and elevated expression levels of immune evasion biomarkers such as PD1 and PD-L1 [51]. Additionally, ccRCC patients with high expression of ADAM12 exhibit lower overall survival rates, with expression positively correlated with PD1, PD-L1, and CTLA4 [52]. In addition to the factors mentioned above, numerous targets associated with PD1 and PD-L1 expression have been reported in previous studies. For instance, EMP3, TGFB1, and TUBB3 are positively correlated with PD-L1 expression [53–55], while APLNR, mean platelet volume, and RBCK1 are negatively correlated with PD-L1 expression [56–58]. These targets have been summarized in Table 2 for better visualization.

**Table 2:** Factors associated with PD1/PD-L1 expression

Factors	PD1/PD-L1	Relevance	Effects	Ref
CCL4	PD1	Positive	Higher immune cell infiltration	[46]
CDKN2A	PD1	Positive	Poor prognosis	[48]
FDX1	PD1	Negative	/	[48]
BMI	PD1	Negative	/	[50]
PRMTs	PD1	Positive	Anticancer effect	[59]
GBP2	PD1	Positive	Carcinogenic effect, immunosuppression	[60]
WAS	PD1	Positive	Immune therapy effect is poor	[61]
NUDT1	PD1	Positive	Poor prognosis	[62]
AGAP2	PD1	Positive	Poor prognosis, immunosuppression	[63]
CXCL13	PD1	Positive	immunosuppression	[64]
CD39	PD1	Positive	immunosuppression	[65]
SLAMF7	PD1	Positive	immunosuppression	[66]
CCL5	PD-L1	Positive	immunosuppression	[45]
CARS	PD-L1	Positive	Poor prognosis	[47]
BMI	PD-L1	Negative	/	[49]
AC084876.1	PD-L1	Positive	Carcinogenic effect	[67]
EMP3	PD-L1	Positive	immunosuppression	[53]
VISTA	PD-L1	Positive	Poor prognosis	[68]
sIL-2R	PD-L1	Positive	Poor response to treatment	[69]
TGFB1	PD-L1	Positive	Poor prognosis	[54]
B7-H3	PD-L1	Positive	Poor prognosis	[70]
HHLA2	PD-L1	Positive	Poor prognosis	[71]
TUBB3	PD-L1	Positive	Carcinogenic effect	[55]
APLNR	PD-L1	Negative	Highly aggressive tumors	[56]
Mean platelet volume	PD-L1	Negative	/	[57]
RBCK1	PD-L1	Negative	Poor prognosis	[58]
Musashi-2	PD-L1	Negative	Better prognosis	[72]
ARNTL2	PD1/PD-L1	Positive	immunosuppression	[51]
ADAM12	PD1/PD-L1	Positive	Poor prognosis	[52]
C1q	PD1/PD-L1	Positive	immunosuppression	[73]

### Specific mechanisms regulating PD1/PD-L1 expression in ccRCC

In the preceding section, we discussed various factors associated with the expression of PD1 and PD-L1 in ccRCC. However, the precise mechanisms governing the regulation and function of PD1/PD-L1 in ccRCC remain an area of active investigation.

**Fig. 1:**
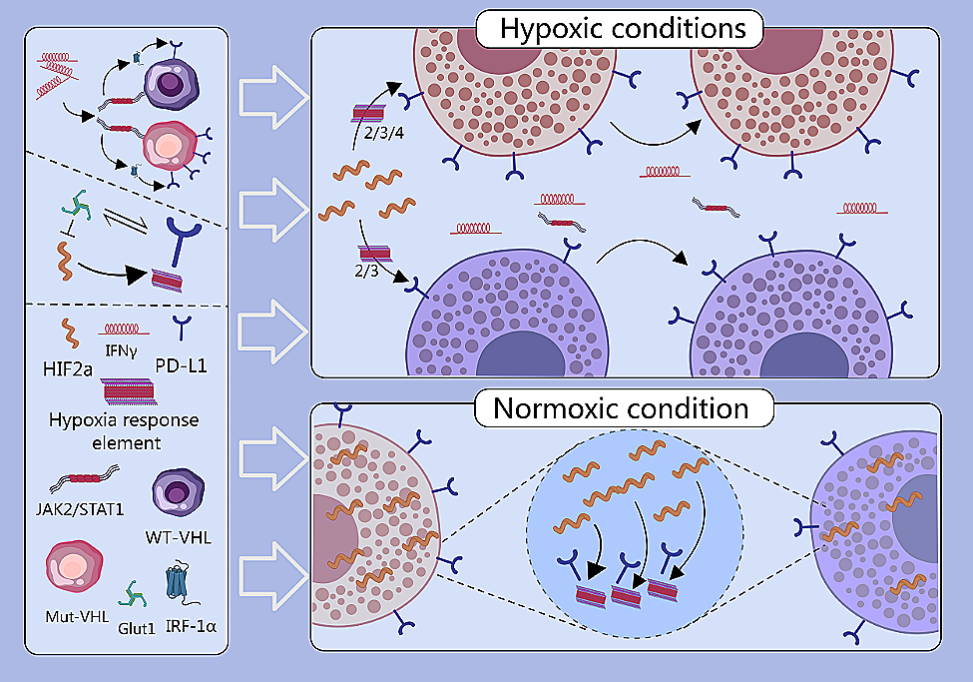
The expression of PD-L1 is co-regulated by IFN-γ and HIF2α. IFN-γ enhances PD-L1 expression through the JAK2/STAT1 signaling pathway, with a more pronounced effect in Mut-VHL cells compared to WT-VHL cells. Additionally, HIF2α directly regulates the transcription of PD-L1 by binding to hypoxia response elements on its promoter, resulting in significantly higher PD-L1 expression in Mut-VHL cells under both normoxic and hypoxic conditions compared to WT-VHL cells

In ccRCC, PD-L1 expression is induced by typical IFN-γ signaling, with significant activation of the IFN-γ pathway observed in tissue samples with high PD-L1 mRNA levels [74]. Additionally, hypoxia-inducible factor 2α (HIF2α) is a critical regulator of PD-L1 expression. HIF2α can directly bind to hypoxia response elements on the PD-L1 promoter, influencing its transcriptional activity [75, 76]. In ccRCC tissues, PD-L1 expression is associated with the expression of JAK2 and STAT1 within the IFN-γ signature. Upon IFN-γ stimulation, both WT-VHL and Mut-VHL ccRCC cells activate the IRF-1α site of the PD-L1 promoter via the JAK2/STAT1 signaling pathway, thereby enhancing PD-L1 expression. Remarkably, the IFN-γ-induced increase in PD-L1 expression is 4.8 times greater in Mut-VHL cells compared to WT-VHL cells. Under normoxic conditions, Mut-VHL cells exhibit significantly higher PD-L1 expression than WT-VHL cells, attributed to elevated basal levels of HIF2α. Conversely, under hypoxic conditions, PD-L1 expression in WT-VHL cells increases up to 1.8 times through activation of hypoxia response elements 2 and 3. In contrast, Mut-VHL cells, already displaying high basal PD-L1 expression, do not further enhance PD-L1 expression under hypoxia despite activation of hypoxia response elements 2, 3, and 4. Overall, Mut-VHL ccRCC cells demonstrate elevated PD-L1 expression due to heightened basal HIF2α levels and a more robust response to IFN-γ stimulation [76]. Furthermore, in ccRCC cell lines where pVHL function is impaired and HIFα cannot be degraded, PD-L1 expression is increased. Restoring pVHL function and silencing HIF2α, rather than HIF1ɑ, decreases PD-L1 expression. In ccRCC cell lines and tissues, there is a strong correlation between the HIF2α-specific target Glut1 and PD-L1 expression [77]. (Fig. 1)

**Fig. 2:**
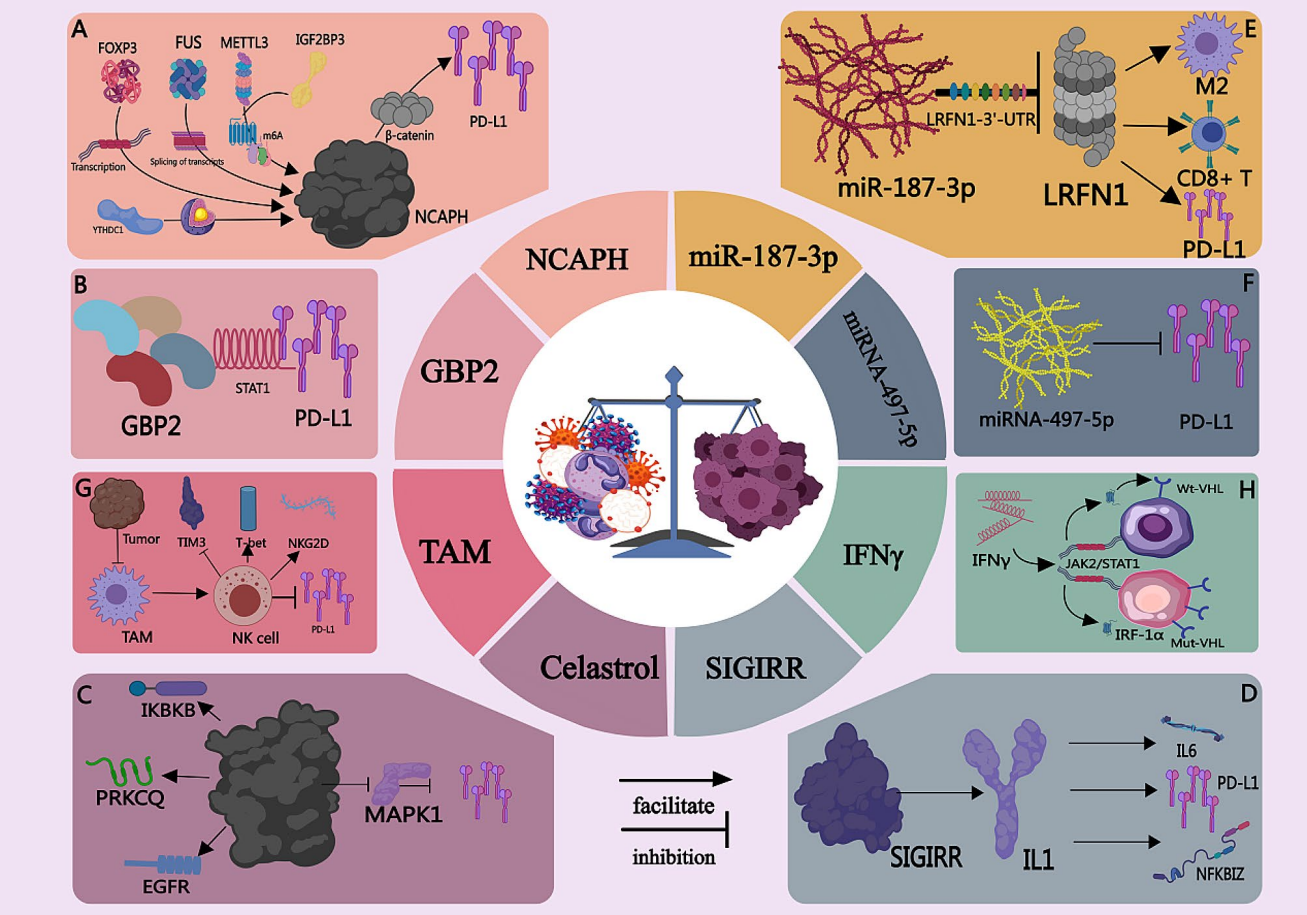
Specific mechanisms regulating PD-1/PD-L1 expression in ccRCC. (**A**) NCAPH increased expression of FOXP3 and FUS mediated transcription splicing and mettl3 mediated m6A modification, driven by the YTHDC1 and IGF2BP3 support, thereby increasing the output and the stability of the mRNA. NCAPH promotes aerobic glycolysis and immune tolerance by inhibiting β-catenin degradation and promoting PD-L1 expression [78]. (**B**) GBP2 regulates the expression of PD-L1 through the STAT1 pathway [79]. (**C**) Celastrol targets EGFR, IKBKB, PRKCQ and MAPK1 at the molecular level, and inhibits the expression of PD-L1 by down-regulating MAPK1 [80]. (**D**) SIGIRR downregulation stimulates activation of the autocrine growth factor IL6, the atypical co-transcription factor NFKBIZ, and the checkpoint inhibitor PD-L1 [81]. (**E**) Up-regulation of miR-187-3p inhibits ccRCC cell proliferation and migration and promotes apoptosis by negatively regulating LRFN1 expression. LRFN1 expression increased M2 macrophage infiltration, CD8 + T cell activity and PD - L1 expression [82]. (**F**) miRNA-497-5p directly targets PD-L1 to inhibit the proliferation, colony formation and migration of ccRCC cells, while promoting cell apoptosis [83]. (**G**) Depletion of TAMs is often accompanied by an increase in tumor-infiltrating NK cells, as indicated by increased expression of T-bet and NKG2D, as well as decreased expression of exhaustion related co-inhibitory molecules PD-1 and TIM-3 [84]. (**H**) IFN-γ enhanced PD-L1 expression through JAK2/STAT1 signaling pathway [76]

In addition to IFN-γ and HIF2ɑ interactions, other factors also regulate PD1/PD-L1 expression (Fig. 2H). NCAPH is elevated in ccRCC and correlates with a poor prognosis. Silencing NCAPH suppresses ccRCC growth in both in vitro and in vivo settings. Its increased expression is regulated through FOXP3-mediated transcription, FUS-mediated transcriptional splicing, and METTL3-mediated m6A modification. Additionally, YTHDC1 facilitates nuclear export of NCAPH mRNA, while IGF2BP3 enhances its mRNA stability in an m6A-dependent manner. NCAPH enhances PD-L1 expression in ccRCC cells by inhibiting β-catenin degradation, thereby promoting aerobic glycolysis and immune tolerance (Fig. 2A) [78]. GBP2 is another adverse prognostic factor in ccRCC that regulates PD-L1 expression through the STAT1 pathway (Fig. 2B) [79]. Celastrol, a key inducer of immunogenic cell death (ICD), targets EGFR, IKBKB, PRKCQ, and MAPK1 at the molecular level and inhibits PD-L1 expression by downregulating MAPK1 (Fig. 2C) [80]. Downregulation of SIGIRR releases IL1 signaling in tumor cells, which subsequently stimulates factors involved in activating inflammatory pathways, including the autocrine growth factor IL6, the unconventional co-transcription factor NFKBIZ, and the immune checkpoint inhibitor PD-L1 (Fig. 2D) [81]. Various microRNAs also participate in regulating the PD1/PD-L1 pathway. For example, upregulation of miR-187-3p inhibits ccRCC cell proliferation, migration, and promotes apoptosis by directly targeting and negatively regulating LRFN1 expression. Increased LRFN1 expression significantly enhances tumor heterogeneity and immune infiltration, characterized by increased M2 macrophage infiltration, CD8 + T cell activity, and PD-L1 expression (Fig. 2E) [82]. Additionally, miRNA-497-5p directly targets PD-L1, inhibiting ccRCC cell proliferation, colony formation, and migration, while promoting apoptosis. Downregulation of miRNA-497-5p leads to upregulation of PD-L1 (Fig. 2F) [83]. Cells within the tumor microenvironment also influence the PD1/PD-L1 pathway. For instance, depletion of TAMs often accompanies an increase in tumor-infiltrating NK cells, which show increased expression of T-bet and NKG2D, and decreased expression of exhaustion-associated co-inhibitory molecules PD1 and TIM-3 (Fig. 2G) [84].

### Mechanisms of the PD1/PD-L1 pathway in ccRCC

**Fig. 3:**
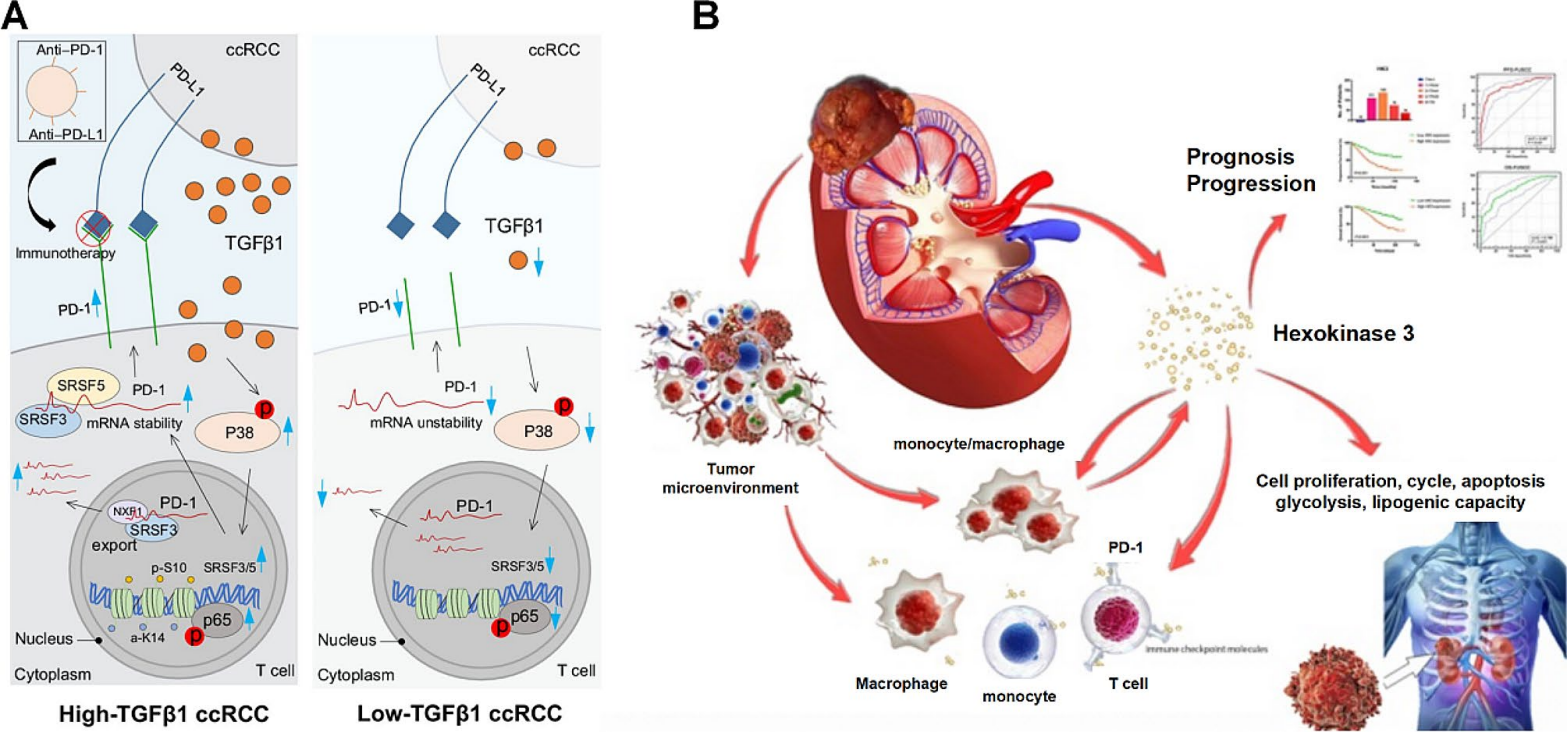
Mechanisms of the PD-1/PD-L1 Pathway in ccRCC. (A) Mechanism by which ccRCC-derived TGFβ1 extends the half-life and mediates the nuclear export of PD-1 mRNA in T cells. Left, TGFβ1 attenuates the antitumor immune activity by prolonging the half-life of PD-1 mRNA and promoting PD-1 mRNA nuclear export in CD8 + T cells. Right, inhibition of TGFβ1 in ccRCC reduces the stability of PD-1 mRNA and decreases extranuclear transport in CD8 + T cells. Blue arrows indicate the increase (up) or decrease (down) of RNA/protein expression, molecular activity, or extranuclear transport. Black arrows indicate the direction and steps of regulation. Red“p”indicates“phosphorylation [85]. Copyright © 2020, American Association for Cancer Research. (**B**) HK3 can influence glycolysis, promote malignant biological processes, and predict the aggressive progression of ccRCC. By stimulating the abundance of infiltrating monocytes/macrophages presenting surface markers, HK3 regulates key molecular subsets of exhausted T-cell immune checkpoint molecules, thereby inducing a microenvironment characterized by active antitumor immune responses [87]. Copyright ©2024, Ivyspring International Publisher

**Fig. 4:**
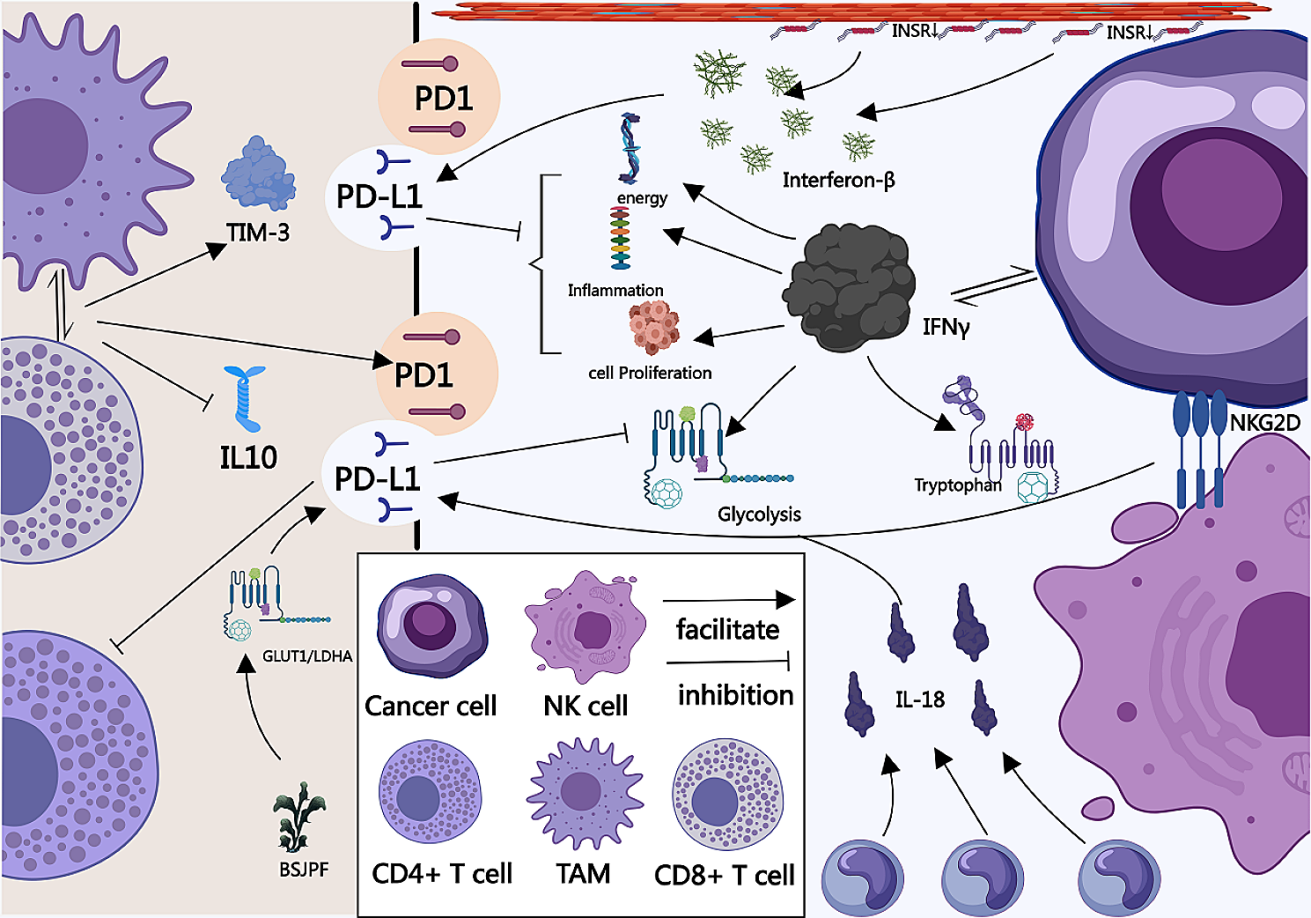
Mechanisms of the PD-1/PD-L1 Pathway in ccRCC. IFN-γ enhances aerobic glycolysis and tryptophan metabolism in ccRCC cells, inducing the transcription of pathways related to inflammation, cell proliferation, and energy metabolism. Silencing PD-L1 can reverse some effects, but tryptophan metabolism and the activation of JAK2 and STAT1 remain unaffected [86]. BSJPF regulates the expression of immune checkpoint molecules PD-L1 and CTLA-4 by enhancing glycolysis related to GLUT1 and LDHA [88]. Recognition of tumor cells through NKG2D induces healthy donor NK cells to express PD-L1, with monocyte-derived IL-18 further upregulating PD-L1 expression and inhibiting CD8 + T cell proliferation in a PD-L1-dependent manner [90]. TAMs can induce an immunosuppressive phenotype in CD4 + T cells by reducing the production of effector cytokines, increasing IL-10 production, and enhancing the expression of co-inhibitory molecules PD-1 and TIM-3 [91]. Endothelial cells with reduced INSR may contribute to axitinib resistance by secreting interferon-β and inducing PD-L1 expression [92]

The specific mechanisms and pathways through which PD1/PD-L1 exert their effects in ccRCC are continuously being explored in greater depth. Research indicates that TGFβ1 derived from ccRCC can activate P38 and induce Ser10 phosphorylation of H3, leading to p65-mediated upregulation of SRSF3 and SRSF5 in T cells. This process extends the half-life of PD1 mRNA in T cells. Additionally, SRSF3, in coordination with NXF1, facilitates the nuclear export of PD1 mRNA in T cells, ultimately restricting their anti-tumor activity (Fig. 3A) [85]. As previously mentioned, IFNγ regulates PD-L1 expression, and PD-L1 expression is integral to mediating IFNγ’s effects. In vitro, IFNγ boosts aerobic glycolysis and tryptophan metabolism in ccRCC cells, stimulating the transcription of pathways linked to inflammation, cell proliferation, and energy metabolism. While these metabolic and transcriptional effects are partly reversed upon temporary PD-L1 silencing, tryptophan metabolism and the activation of Jak2 and STAT1 remain unaffected (Fig. 4) [86]. Studies have shown that HK3 also regulates glycolysis and the malignant behavior of ccRCC cells. HK3 expression is significantly increased in ccRCC tissues, predicting tumor progression and poor prognosis. Moreover, HK3 stimulates monocyte/macrophage infiltration and regulates the expression of immune checkpoint molecules PD1 and CTLA-4 in exhausted T cells, thereby inhibiting immune escape of tumor cells (Fig. 3B) [87]. Traditional Chinese medicine, Bu Shen Jian Pi Fang (BSJPF), has demonstrated the ability to suppress tumor proliferation and modify the immune-exclusion status of the tumor microenvironment. It achieves this by enhancing GLUT1- and LDHA-associated glycolysis and regulating the expression of immune checkpoint molecules such as PD-L1 and CTLA-4 (Fig. 4) [88]. In ccRCC, PD-L1 positivity is closely associated with increased lymphocyte infiltration around the tumor [89]. There is a high frequency of PD-L1 + tumor-infiltrating NK cells in ccRCC. Recognition of tumor cells through NKG2D can induce PD-L1 expression in healthy donor NK cells, and IL-18 derived from monocytes can further upregulate PD-L1 expression. PD-L1 high-expressing NK cells exhibit an activated phenotype and enhanced effector function but simultaneously inhibit CD8 + T cell proliferation in a PD-L1-dependent manner (Fig. 4) [90]. TAMs and isolated TAMs within the tumor microenvironment can induce a more immunosuppressive phenotype in CD4 + T cells by reducing effector cytokine production, increasing IL-10 production, and enhancing the expression of co-inhibitory molecules PD1 and TIM-3 (Fig. 4) [91]. Furthermore, endothelial cells exhibiting decreased INSR levels in RCC can secrete interferon-β, triggering PD-L1 expression and thereby contributing to axitinib resistance (Fig. 4) [92].

Understanding these regulatory mechanisms is crucial for developing effective therapeutic strategies targeting the PD1/PD-L1 pathway, which holds significant promise for improving outcomes in patients with ccRCC.

### Prognostic value of PD1/PD-L1 expression in ccRCC

As with other malignancies, the expression levels of PD1 and PD-L1 in ccRCC are intricately associated with the tumor’s prognosis, influencing various clinical outcomes. Relevant studies have shown that higher expression levels of PD1 and PD-L1 are significantly associated with clinicopathological features such as primary tumor staging, regional lymph node involvement, distant metastasis, nuclear grade, and histological tumor necrosis [24, 93–96]. The presence of PD1 in TILs correlates significantly with worse disease-free survival (DFS) and disease-specific survival (DSS) [97]. However, some studies indicate that PD1 expression in TILs is not associated with the risk of death or recurrence [98]. High PD-L1 expression in ccRCC is associated with poorer overall survival (OS), as well as decreased DFS, progression-free survival (PFS), and DSS [24, 93–95, 99, 100]. In ccRCC, high PD-L1 expression is often accompanied by increased infiltration of exhausted CD8 + T cells, FOXP3 positive TILs, and elevated expression of VEGF within the tumor [101–104]. Research indicates that patients with PD-L1-positive tumors are 2.9 times more likely to die within five years post-nephrectomy compared to those with less than 5% PD-L1 positivity in tumor cells. In ccRCC patients with lung metastases, those with high densities of PD1 positive lymphocytes and PD-L1 and/or PD-L2 positive tumor cells in the metastatic lesions have a poorer prognosis, with a 3.1-fold increased risk of death after metastasectomy compared to negative patients [105]. Plasma PD-L1 is also an important prognostic marker, with concentrations exceeding 793 ng/mL significantly associated with decreased survival [24]. Additionally, co-expression of HHLA2/PD-L1 predicts an increased risk of progression and death [106].

## Clinical efficacy and safety of PD1/PD-L1 inhibitors in ccRCC

Currently, PD1/PD-L1 inhibitors have become an important class of immunotherapy in the treatment of ccRCC. Their clinical efficacy has been validated in multiple clinical trials. In this section, we will summarize the main clinical trial data of PD1/PD-L1 inhibitors in the treatment of ccRCC, including the trial results of drugs such as Nivolumab and Pembrolizumab. We will explore the efficacy of PD1/PD-L1 inhibitors, common resistance mechanisms, and strategies to address resistance.

### Nivolumab

Nivolumab is a PD1 inhibitor. The CheckMate 374 study validated the safety and efficacy of Nivolumab monotherapy at a dose of 240 mg every two weeks for previously treated advanced ccRCC patients. Patients exhibited fewer major adverse events and relatively higher objective response rates, with moderate progression-free survival and overall survival [107]. 

The HCRN GU16-260-Cohort, a Phase II Study, demonstrated efficacy of Nivolumab monotherapy in treatment-naive ccRCC patients, particularly showing significant effects in patients with PD-L1 expression above 20%, while patients with 0% PD-L1 expression showed poorer responses. Although the efficacy in intermediate- and high-risk patients was lower compared to Nivolumab/ipilimumab combination therapy, low-risk patients significantly benefited from Nivolumab monotherapy. Rescue therapy with Nivolumab/ipilimumab for patients unresponsive to Nivolumab monotherapy showed limited efficacy and feasibility [108].

The GETUG-AFU 26 NIVOREN Phase II trial evaluated the efficacy and safety of Nivolumab in patients with metastatic ccRCC who had experienced treatment failure with antiangiogenic therapies, including 73 patients with brain metastases [109]. Results revealed a 12% intracranial response rate in untreated patients, while patients with multiple or larger than 1 cm brain lesions showed no objective response. The median intracranial progression-free survival was 2.7 months and 4.8 months in untreated and treated groups, respectively, with 12-month overall survival rates of 67% and 59%. Most untreated patients (72%) required subsequent local brain therapy. Despite good tolerability with no unexpected toxicity, Nivolumab showed limited activity in ccRCC brain metastases patients, emphasizing the importance of brain imaging and local therapy before immune checkpoint inhibitors. Additionally, a study analyzed AXL expression in tumor specimens of metastatic ccRCC patients treated with Nivolumab following antiangiogenic therapy failure, revealing that high AXL expression correlated with lower treatment response rates and shorter progression-free survival. AXL expression was closely associated with tumor PD-L1 expression, particularly in tumors with VHL gene inactivation [110]. In a translational study, specific baseline circulating immune cell populations and soluble factors predictive of clinical outcomes were identified in metastatic ccRCC patients treated with Nivolumab. Responders showed enrichment of baseline unswitched memory B cells (NSwM B cells), which correlated with improved overall survival (OS) and progression-free survival (PFS). Additionally, responders exhibited increased levels of circulating T follicular helper cells (Tfh) and tertiary lymphoid structures (TLS). NSwM B cells positively correlated with Tfh, TLS, and tumor center CD20 + B cells, but inversely correlated with BCA-1/CXCL13 and BAFF, both of which were significantly associated with poorer OS [111].

A phase 1–2 trial demonstrated that the tyrosine kinase inhibitor sitravatinib, in combination with Nivolumab, enhanced response to anti-PD1 therapy in advanced ccRCC patients following progression on antiangiogenic therapy, with safe and effective outcomes, suggesting a rational combination therapy strategy for late-stage ccRCC patients [19]. Furthermore, a single-arm, interventional, phase 2 study in locally advanced ccRCC patients undergoing radical nephrectomy received sitravatinib in combination with neoadjuvant Nivolumab. Results showed no significant improvement in overall response rate with short-term neoadjuvant sitravatinib and Nivolumab therapy [112].

A phase 1b trial suggested that the oral CXCR4 inhibitor Mavorixafor, in combination with Nivolumab, exhibited potential anti-tumor activity in metastatic ccRCC patients unresponsive to Nivolumab monotherapy, particularly evident in patients with stable disease [113].

The CheckMate 009 trial demonstrated the improvement in survival of partial ccRCC patients with Nivolumab treatment. Molecular characterization of Nivolumab response through paired lesion biopsies in the CheckMate 009 trial revealed an association between treatment response and T cell infiltration, independent of TCR clonality, suggesting potential mechanisms of Nivolumab resistance in non-responders, including RIG-I-MDA5 pathway involvement. Additionally, a molecular subtype was identified that exhibited good response to Nivolumab but resistance to sunitinib [114].

### Pembrolizumab

Pembrolizumab is an immune checkpoint inhibitor that targets the PD1 receptor. An open-label, single-arm phase II trial involving 110 untreated patients with advanced ccRCC was conducted in a study. revealing significant antitumor activity of pembrolizumab monotherapy in this patient cohort. The results showed that 36.4% of patients achieved objective responses, including 3.6% complete responses and 32.7% partial responses, with a disease control rate of 58.2%. Most patients (68.2%) experienced shrinkage in target lesions, with 30.9% showing reductions of 60% or more. For responders, the median duration of response was 18.9 months, with 64.1% of responses lasting 12 months or longer. The study reported a median progression-free survival of 7.1 months. Median overall survival was not reached, with corresponding 12-month and 24-month overall survival rates of 88.2% and 70.8%, respectively. Additionally, durable treatment effects were observed across various risk groups. Notably, 30.0% of patients reported grade 3–5 treatment-related adverse events, with colitis and diarrhea being the most common. These findings suggest that pembrolizumab monotherapy as a first-line treatment option for advanced ccRCC exhibits durable treatment effects and good safety profile [115].

The RAPPORT trial assessed the safety and efficacy of short-course pembrolizumab following total metastatic irradiation in patients with oligometastatic ccRCC. The results demonstrated excellent tolerability of this treatment regimen among patients, with outstanding local control effects. The freedom from local progression rate was 92%, with 63% of patients experiencing partial responses and 83% achieving disease control. The study showed 1-year and 2-year overall survival rates of 90% and 74%, respectively, along with progression-free survival rates of 60% and 45%, respectively. Although the single-arm design and patient selection limit interpretation, these promising findings justify further investigation [116].

### Atezolizumab

Atezolizumab is an immune checkpoint inhibitor targeting PD-L1. The COSMIC-021 trial assessed the effectiveness of combining cabozantinib with atezolizumab in patients diagnosed with advanced RCC, including both clear cell and non-clear cell subtypes. The results showed that in patients with advanced ccRCC, the objective response rates were 53% and 58% in the 40 mg and 60 mg dose groups, respectively, with 3% and 11% achieving complete response. The study reported median progression-free survivals of 19.5 months and 15.1 months, respectively. Among patients with nccRCC, the objective response rate was 31%, comprising entirely partial responses, with a median progression-free survival of 9.5 months. Grade 3 or 4 treatment-related adverse events were observed in 71% and 67% of patients in the 40 mg and 60 mg ccRCC groups, respectively, and in 38% of patients in the nccRCC group. Treatment-related adverse events necessitated discontinuation of both agents in 15%, 6%, and 3% of patients, respectively. These findings underscore the promising clinical efficacy and tolerability of this novel combination therapy in patients with advanced ccRCC and nccRCC [117].

In a phase Ia study, 70 patients with metastatic RCC received intravenous atezolizumab every 3 weeks. Grade 3 treatment-related adverse events were observed in 17% of patients, while immune-mediated adverse events of the same grade occurred in 4%, with no instances of grade 4 or 5 events reported. Overall survival was evaluated in 63 ccRCC patients with a median of 28.9 months, and progression-free survival with a median of 5.6 months. The objective response rate was 15% in 62 evaluable patients. Objective response rate was assessed based on PD-L1 expression. For patients with Fuhrman grade 4 and/or sarcomatoid histology, the objective response rate was 22%. Reductions in circulating plasma markers and acute-phase proteins, coupled with an elevated baseline ratio of effector T-cell to regulatory T-cell gene expression, were associated with response to atezolizumab. These findings underscore the manageable safety profile and encouraging antitumor efficacy of atezolizumab in metastatic RCC patients [118].

### Durvalumab

Durvalumab is a kind of PD-L1 inhibitors. In a phase Ib/II study investigating guadecitabine and durvalumab for advanced ccRCC, 57 patients received guadecitabine and durvalumab. The study comprised 6 phase Ib and 51 phase II patients, with 36 patients in Cohort 1 being checkpoint inhibitor (CPI) treatment-naive with no prior therapy and 15 patients in Cohort 2 receiving up to two prior systemic treatments, including one CPI. The combination of guadecitabine 45 mg/m2 and durvalumab 1500 mg was deemed safe. The primary objective of Cohort 1 was an overall response rate (ORR) of 22%, with 16 patients (44%) experiencing stable disease (SD). In Cohort 1, the median PFS was 14.26 months, while in Cohort 2, it was 3.91 months. Median OS was not reached. In Cohort 2, one patient achieved a partial response, and 60% achieved SD. The most frequent adverse event was asymptomatic neutropenia. Although Cohort 1 did not meet its primary objective, the tolerability and PFS outcomes in CPI treatment-naive patients suggest a need for further investigation [119].

We summarize the clinical trials of PD1/PD-L1 inhibitors in ccRCC in Table 3 for a more intuitive presentation.

**Table 3:** Clinical trial of anti-PD1/PD-L1 in ccRCC

Drugs	Targets	Combination	Effects	ClinicalTrials.gov	Ref
Nivolumab	PD1	/	It is safe and effective for patients with previously treated advanced ccRCC.	NCT02596035	[107]
Nivolumab	PD1	/	A significant effect was observed in patients with PD-L1 expression higher than 20%.	NCT03117309	[108]
Nivolumab	PD1	/	There is limited efficacy in untreated ccRCC patients with brain metastases.	NCT03013335	[109]
Nivolumab	PD1	/	Higher levels of AXL expression were associated with lower treatment response rates and shorter progression-free survival.	NCT03013335	[110]
Nivolumab	PD1	/	Specific circulating immune cell populations and/or soluble factors at baseline were identified to predict clinical response to nivolumab treatment in m-ccRCC patients.	NCT03013335	[111]
Nivolumab	PD1	sitravatinib	The combination of sitravatinib and nivolumab is safe and effective, with mild side effects, and is a reasonable combination strategy for patients with advanced ccRCC.	NCT03015740	[19]
Nivolumab	PD1	sitravatinib	Short-term combination therapy with sitravatinib and nivolumab did not significantly improve ORR in patients with locally advanced ccRCC undergoing radical nephrectomy.	NCT03680521	[112]
Nivolumab	PD1	Mavorixafor	Mavorixafor in combination with Nivolumab has shown potential antitumor activity in patients with metastatic ccRCC who did not respond to Nivolumab monotherapy.	NCT02923531	[113]
Nivolumab	PD1	/	Molecular signatures predicting response to nivolumab.	NCT01358721	[114]
Pembrolizumab	PD1	/	Single-agent Pembrolizumab showed durable efficacy and good safety in the treatment of advanced ccRCC.	NCT02853344	[115]
Pembrolizumab	PD1	Radiotherapy	It is well tolerated and has excellent local control.	NCT02855203	[116]
Atezolizumab	PD-L1	Cabozantinib	Combination therapy showed good clinical activity, and has an acceptable tolerance.	NCT03170960	[117]
Atezolizumab	PD-L1	/	It has shown a manageable safety profile and promising antitumor activity in patients with metastatic RCC.	NCT01375842	[118]
Durvalumab	PD-L1	Guadecitabine	Combination therapy is safe and effective.	NCT03308396	[119]

## Combination therapy strategies with PD1/PD-L1 inhibitors in ccRCC

As summarized above, although PD1/PD-L1 inhibitors show some potential in the treatment of ccRCC, their limitations are still evident, including low response rates, development of drug resistance, tumor heterogeneity, immune escape mechanisms, insufficient persistence of efficacy, side effects, and lack of predictive biomarkers.With the deepening of our understanding of tumor immune escape mechanisms, more and more research has focused on the combined application of PD1/PD-L1 inhibitors with other therapeutic modalities.The rationale for combining PD1/PD-L1 inhibitors with other therapies is to enhance the immune response, overcome immune escape mechanisms, target different tumor biological characteristics, and reduce side effects.PD1/PD-L1 inhibitors enhance T cell activity by blocking the inhibitory pathway, and this effect can be further enhanced when combined with chemotherapy or radiotherapy that increases tumor antigen release. Co-inhibition with other immune checkpoints such as CTLA-4 or modification of the tumor microenvironment through anti-angiogenic agents can enhance the immune response. Targeted therapies can address specific tumor drivers while working synergistically with immune activation, and cell therapies such as CAR T cells with sustained activity under PD1/PD-L1 block also benefit. In addition, combination therapy can reduce the dose of a single therapy, thereby potentially reducing adverse effects while maintaining efficacy.Together, these combination therapies are designed to further increase patient response rates, prolong patient survival, and improve treatment outcomes. In this section, we will focus on the strategy of combining PD1/PD-L1 immune checkpoint inhibitors with other therapeutic methods in the treatment of ccRCC, and analyze the clinical efficacy and safety, so as to provide reference for future clinical practice.

**Fig. 5:**
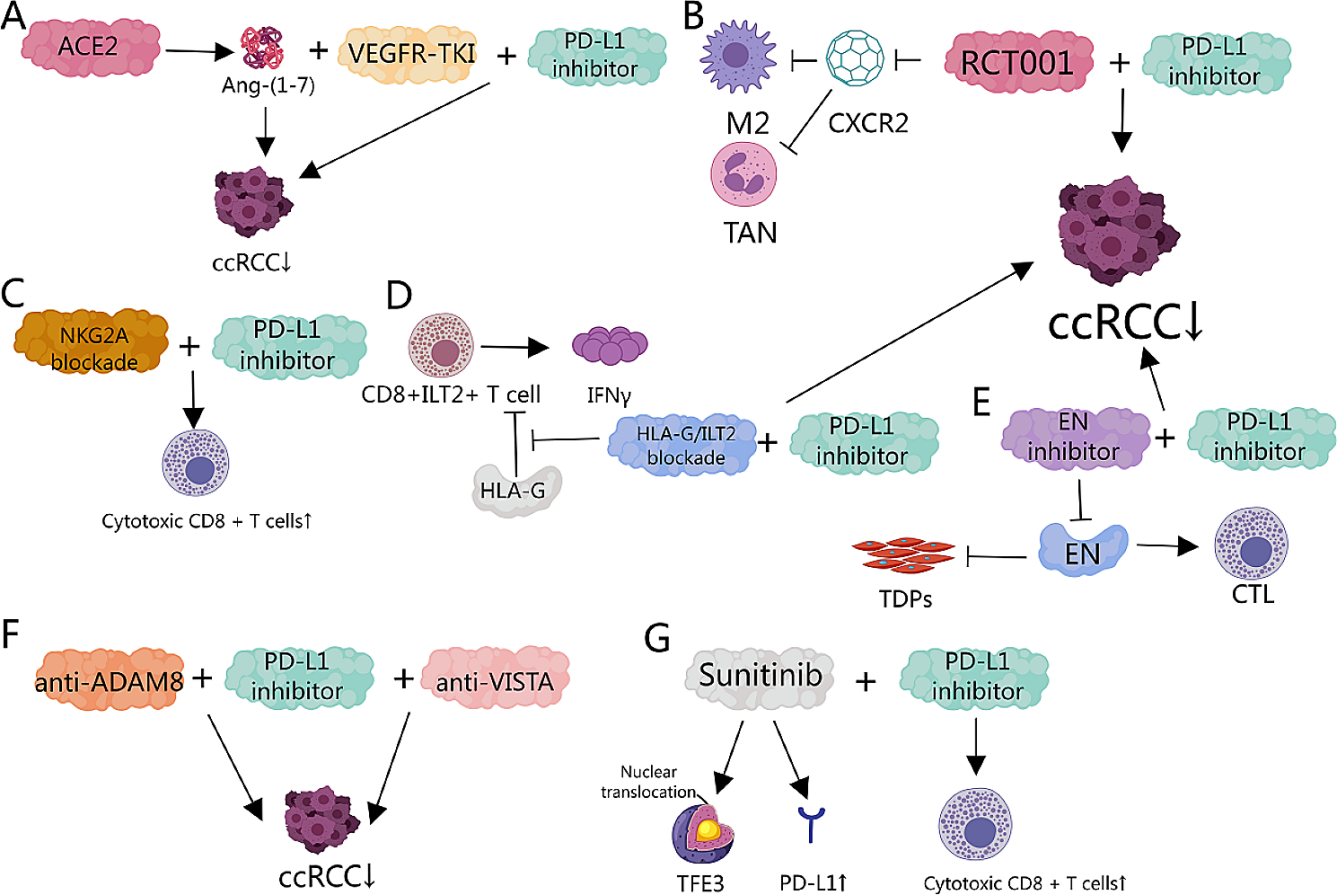
Combination Therapy Strategies with PD-1/PD-L1 Inhibitors in ccRCC. (**A**) Ang-(1–7) mediated ACE2 to inhibit tumor proliferation. Treatment of ccRCC xenografts with VEGFR-TKIs reduced ACE2 expression. The addition of Ang-(1–7) to PD-L1 pathway inhibitors and VEGFR-TKIs further inhibited tumor growth [121]. (**B**) The CXCR2 inhibitor RCT001 enhanced the efficacy of anti-CTLA4 + anti-PD1 therapy by inhibiting tumor-associated M2 macrophages and tumor-associated neutrophils [122]. (**C**) NKG2A + CD8 + T cells exhibit impaired effector function and tissue resident characteristics. The combination of PD-1 and NKG2A blockade can enhance the ability of CD8 + T cell effector function reactivation [123]. (**D**) The expression of HLA-G on target cells specifically inhibited the cytotoxic effect of CD8 + ILT2 + T cells independent of PD-1 + TILs, and blockade of HLA-G/ILT2 interaction antagonized this effect. These targets can be combined with anti-PD1 /PD-L1 therapies [124]. (**E**) High EN expression was negatively correlated with CTL in ccRCC tissues. Combined application of anti-EN and anti-PD1 antibodies significantly enhanced the antitumor effect [125]. (**F**) Anti - ADAM8 or anti - VISTA can improve the effectiveness of anti-PD1 treatment [126, 127]. (**G**) Sunitinib can induce TFE3 nuclear translocation and increase PD-L1 expression in ccRCC. Sunitinib combined with PD-L1 inhibitor significantly enhanced the cytotoxic activity of CD8 + cells, thereby inhibiting the growth of ccRCC [120]

As previously discussed, the combination of PD1/PD-L1 inhibitors with receptor tyrosine kinase inhibitors (RTKIs) has been shown to significantly improve outcomes in patients with ccRCC. Notably, the combination of sitravatinib with nivolumab demonstrated improved outcomes in ccRCC patients who progressed after anti-angiogenic therapy [19]. However, it is important to note that the use of sitravatinib combined with nivolumab as neoadjuvant therapy before radical nephrectomy in patients with locally advanced ccRCC did not significantly improve the ORR [112]. Additionally, the combination of cabozantinib and atezolizumab has shown promising efficacy in advanced ccRCC [117]. Sunitinib, another RTKI, has been found to cause TFE3 nuclear translocation and increased PD-L1 expression in ccRCC. The combination of sunitinib with PD-L1 inhibitors significantly enhances CD8 + cytotoxic activity, thereby inhibiting ccRCC growth (Fig. 5G) [120]. Moreover, higher expression levels of angiotensin-converting enzyme 2 (ACE2) in ccRCC patients are associated with better overall survival. In vitro and preclinical models of ccRCC have shown that ACE2 inhibits tumor proliferation, with the heptapeptide angiotensin (Ang-(1–7)) potentially mediating this effect. Treatment of ccRCC xenografts with VEGFR-TKIs reduces ACE2 expression, while the combination of VEGFR-TKIs and Ang-(1–7) produces an additive inhibitory effect on tumor growth and improves survival outcomes. Finally, in immunocompetent RCC models, the addition of Ang-(1–7) to PD-L1 pathway inhibitors and VEGFR-TKIs further inhibited tumor growth (Fig. 5A) [121].

In addition to the clinical efficacy demonstrated by the combination of tyrosine kinase inhibitors, the oral CXCR4 inhibitor mavorixafor combined with nivolumab has shown potential antitumor activity in clinical trials for metastatic ccRCC patients who did not respond to nivolumab monotherapy, particularly in patients with stable disease [113]. Furthermore, RCT001, an optimized CXCR2 inhibitor, enhances the efficacy of anti-CTLA4 + anti-PD1 therapies by inhibiting tumor-associated M2 macrophages and tumor-associated neutrophils. Similar efficacy has been observed in human ccRCC tumors when RCT001 is combined with anti-PD1 treatment (Fig. 5B) [122].

More potential targets are continually being developed to enhance the efficacy of PD1/PD-L1 inhibitors in treating ccRCC. High infiltration of NKG2A + CD8 + T cells in ccRCC correlates with reduced overall survival and resistance to immunotherapy. These cells display increased expression of checkpoint molecules, impaired effector function, and characteristics typical of tissue-resident cells. Combined blockade of PD1 and NKG2A has demonstrated improved efficacy in restoring CD8 + T cell effector functions (Fig. 5C) [123]. There are significant differences between CD8 + ILT2 + and CD8 + PD1 + TIL subpopulations. Despite sometimes appearing at similar frequencies within the TIL population, these subpopulations show little phenotypic overlap. CD8 + ILT2 + T cells demonstrate heightened cytotoxicity and IFNγ production. Target cells expressing HLA-G specifically suppress the cytotoxic activity of CD8 + ILT2 + T cells, which can be reversed by blocking HLA-G/ILT2 interactions. Consequently, CD8 + ILT2 + TILs may serve as a valuable pool of fully differentiated cytotoxic T cells in the tumor microenvironment. Unlike PD1 + TILs, their function is specifically restrained by HLA-G. They could be targeted alongside anti-PD1/PD-L1 therapies or in cases where anti-PD1/PD-L1 is ineffective (Fig. 5D) [124]. High expression of endothelium-derived neurotrophic factor (EN) is negatively correlated with cytotoxic T lymphocyte (CTL) infiltration in ccRCC tissues. High EN expression signifies an activated state of vascular smooth muscle cells, and inhibiting EN significantly increases CTL infiltration in mice. The combined application of anti-EN and anti-PD1 antibodies significantly enhances antitumor effects (Fig. 5E) [125]. Additionally, studies have shown that ADAM8 promotes proliferation, migration, and invasion of ccRCC cells in vitro. Silencing ADAM8 can inhibit tumor formation in ccRCC cells and improve the effectiveness of anti-PD1 therapy (Fig. 5F) [126]. Furthermore, VISTA is selectively overexpressed in ccRCC tumors. The combined blockade of VISTA and PD1 has produced synergistic effects in 20% of RCC patients, suggesting that this combination therapy is a promising strategy (Fig. 5F) [127].

Immune checkpoint inhibitors can exhibit resistance when used as monotherapies. Recent studies indicate that dual immune checkpoint inhibition also shows promising efficacy in treating ccRCC. LAG-3 is overexpressed in ccRCC and its expression is significantly correlated with PD-L1 expression. RCCs that are LAG-3 + and PD-L1 + are associated with higher TNM staging and higher Fuhrman nuclear grades, and PD-L1+/LAG-3 + RCCs demonstrate poorer cancer-specific survival (CSS). Multivariate analysis suggests that PD-L1+/LAG-3 + mRCC is an adverse prognostic factor for CSS. These findings suggest that the combined blockade of LAG-3 and PD1/PD-L1 could be a potential therapeutic strategy for RCC [128]. Additionally, a study reported a case involving a 60-year-old female patient with locally advanced renal cancer (T3aN0M0), who developed a palatal lesion resembling a vascular tumor. Histopathological examination following excisional biopsy confirmed that the lesion was an oral metastasis originating from ccRCC. The patient was treated with a combination of two immune checkpoint inhibitors (ICIs), specifically the PD1 inhibitor nivolumab and the CTLA-4 inhibitor ipilimumab. After three cycles of systemic immunotherapy, the patient’s palatal lesion completely healed, and there was no recurrence observed during the 13-month follow-up period [129].

In addition to the aforementioned combination strategies, prior study have also demonstrated the enhanced efficacy of combining immune checkpoint inhibitors with radiotherapy in the treatment of ccRCC [116]. In summary, as our understanding of tumor immune evasion mechanisms deepens, more research will explore increasingly effective combination approaches, aiming to further improve patient response rates, extend survival times, and enhance overall treatment outcomes.

## Predictive and resistance factors for PD1/PD-L1 inhibition in ccRCC

Research indicates that the clinical benefits of PD1/PD-L1 inhibitors are associated with the loss of function and mutations in the PBRM1 gene [15]. Using the MutFormer model to classify PBRM1 mutations into functional loss or normal function groups, and combining genomic and transcriptomic data to construct a comprehensive PBRM1 score, can effectively predict patients’ response to anti-PD1 therapy [130]. It is noteworthy that the proportion of PBRM1 mutations in the high m6A score group is significantly lower than that in the low m6A score group, and a low m6A score may indicate better treatment response and prognosis [131]. Furthermore, PARP1 can serve as a biomarker for predicting the response of ccRCC patients with PBRM1 mutations to ICI therapy. The OS of the PARP1 low-expression group is significantly higher than that of the PARP1 high-expression group [132].

Research has analyzed the TME of intermediate- to high-risk ccRCC patients using spatial transcriptome sequencing (ST-seq) and found that a high-level TME is rich in exhausted/pro-tumor immune cells and lacks specific checkpoint gene expression, suggesting its immunogenicity but unfavorable response to ICIs therapy. The types and status of immune cells infiltrating tumors may help predict responses to ICI therapy [133]. In ccRCC patients responding to ICI therapy, higher densities of tumor-infiltrating T cells, CTLs, and PD1-positive immune cells are observed [134]. However, studies have also observed that compared to non-responders, responders to ICI have lower frequencies of CD8 + tumor-infiltrating T cells and TLS, as well as reduced PD-L1 expression [135]. These potentially conflicting results may be due to sample size limitations. Neutrophils in ccRCC can also resist anti-cancer immune therapy with nivolumab and pembrolizumab through low/no expression of CTLA-4, PD1, and PD-L1 [136]. PD1 expression on Tregs is associated with resistance to PD1 blockade in mccRCC, suggesting a synergistic effect of targeting Tregs with PD1 inhibitors [137]. In localized ccRCC, infiltration of exhausted TILs (CD8 + PD1 + Tim-3 + Lag-3+) and ICOS + Tregs signifies poor prognosis, but these patients may benefit from adjunctive therapy with TME modulators and checkpoint blockade [138]. A study observed that a neutrophil-to-lymphocyte ratio (NLR) ≥ 3, low hemoglobin, and high platelet count at 12 weeks after initiation of ICI therapy have negative predictive and prognostic effects in metastatic RCC patients. Normalization of NLR in baseline-elevated patients is associated with longer median OS and treatment response [139]. It is worth mentioning that the presence of TLS, especially intratumoral and secondary follicle-like TLS, is significantly associated with better survival and objective response rates in ccRCC patients receiving anti-PD1/PD-L1 immunotherapy [140].

Previous studies have constructed risk assessment models based on specific gene sets to predict the efficacy of PD1/PD-L1 inhibitors. A study has identified two molecular subtypes distinguished by clustering of necrosis-related genes. Cluster 1 shows activation of classical oncogenic pathways, whereas Cluster 2 exhibits activation of immune-related pathways. Patients in Cluster 2 respond better to anti-PD1 inhibitor therapy [141]. Researchers have developed a model called Immune Exclusion Score (IES) to predict the efficacy of anti-PD-(L)1 immunotherapy. Patients with low IES exhibit longer progression-free survival and better treatment response [142]. Another study constructed a ubiquitin score to assess the ubiquitination outcome of individual patients, finding that the high-score group is associated with higher levels of immune cell infiltration and expression of PD1/PD-L1/CTLA-4, possibly indicating sensitivity to PD1 therapy [143]. Prognostic markers associated with disulfide death show that patients in the high-risk group have a more complex tumor immune microenvironment, making them more susceptible to tumor immune escape during immunotherapy, while patients in the low-risk group have better prognosis and response to immune therapy, especially to anti-PD1 and anti-CTLA-4 inhibitors [144]. A three-gene model based on CFB, PPP1R18, and TOM1L1 divides patients into high-risk and low-risk groups. Although the high-risk group shows lower infiltration of RMCs, higher tumor mutation burden (TMB), and worse prognosis, they are more sensitive to immune therapy with anti-PD1 and anti-CTLA-4, while the low-risk group responds better to immune therapy with anti-PD-L1 [44]. A risk scoring model based on differential expression genes in the complement and coagulation cascades pathway (CCCP) observes that patients in the low-risk group have better responsiveness to ICIs and longer OS [145]. A signature based on SHC1, IRF7, KDR, JAK3, and CXCL5 shows that in high-risk patients, there is a higher relative abundance of TFH cells, regulatory T cells, and M0 macrophages in tumors, as well as higher expression of PD1, CTLA-4, LAG3, and CD47, suggesting potential benefits from immune checkpoint inhibitor therapy [146]. Another study established an ERV signature based on nine ERV expression patterns, showing that patients in the low-risk group have better survival outcomes with nivolumab treatment [147]. A signature composed of PML, CDKN2B, COL1A2, CHRDL1, HPGD, CGN, and TGFBR3 indicates that patients in the high-risk group benefit more from immune therapy [148]. A gene signature based on FOXM1, TOP2A, KIF18B, and NUF2 shows that patients in the low-risk group have a stronger response to anti-PD1 immune therapy, and PBRM1 mutation is associated with the gene signature [149]. A four-gene risk assessment model involving CLDN4, SEMA3G, CAT, and UCN suggests that the high-risk group may exhibit higher sensitivity to immune therapy checkpoint inhibitors PD1, CTLA-4, IL-6, and LAG3 in ccRCC patients [150]. There is also research on constructing a risk scoring model based on 13 co-stimulatory molecular genes associated with prognosis. The results show that the high-risk subgroup tends to have higher cytotoxic activity scores and immune phenotype scores for CTLA4 and PD1/PD-L1/PD-L2 blockade inhibitors, indicating that these patients may be more suitable for immune therapy [151].

Some single factors can also predict the efficacy of PD1/PD-L1. Genes that can predict efficacy include NF2, TSC1, RUFY4, CD3, CTSZ, LGALS1, AXL, TNFSF13B, TNFRSF9, DDX39BCYT, and HIF1A, among others [110, 152–161]. In addition, intratumor heterogeneity (ITH), tumor VHL status, CTLA4 methylation levels, age, chromosomal changes, etc., are also important factors for predicting PD1/PD-L1 efficacy [156, 162–165]. Human endogenous retroviruses (hERVs) are also potential factors for predicting the efficacy of PD1/PD-L1 inhibitors, among which the expression of hERV 4700 is associated with a better response in ccRCC patients receiving aPD1 therapy [166]. In patients responding to PD1/PD-L1 blockade agents, the expression of ERV3-2 in tumors is significantly higher than in non-responders [167]. Furthermore, decitabine, a demethylating agent, has been shown to induce the expression of transposable elements (TEs) such as LINE1 and ERVs (ERV3-2 and ERV4700), along with antiviral signaling. This effect may augment the response of kidney cancer cell lines and primary cells to ICB [168]. Current frontier research also explores computer vision models that, by combining tumor inherent spatial microheterogeneity and TIL features, can capture meaningful characterizations that selectively respond to ICI in predicting responses of ccRCC patients to aPD1 treatment [169].

Finally, it is worth mentioning that although PD1/PD-L1 expression is the most direct factor for predicting PD1/PD-L1 inhibitor efficacy, the variability in PD-L1 expression across ccRCC indicates that assessing it as a predictive biomarker for PD1 blockade may necessitate analysis of metastatic sites. Furthermore, given that PD-L1 expression predominantly occurs in areas with high nuclear grading, it is crucial to selectively target these regions during evaluation to prevent false-negative outcomes [170].

## Summary and prospect

As precision medicine advances, personalized treatment strategies for ccRCC are gaining increasing attention. Tailoring treatment plans based on patients’ molecular profiles, immune status, and other clinical parameters can significantly enhance the efficacy of PD1/PD-L1 inhibitors. Future research should focus on identifying and validating biomarkers to predict patients’ responses and prognoses with PD1/PD-L1 inhibitors. For instance, TMB, microsatellite instability (MSI), and specific gene mutations (such as PBRM1 and VHL) could serve as indicators of immunotherapy response [171–173]. Personalized treatment must consider not only genetic mutations but also a comprehensive assessment of the patient’s immune status. Immune monitoring techniques, such as flow cytometry, immunohistochemistry, and high-throughput sequencing, can evaluate the status and function of immune cells within patients, aiding in predicting treatment responses and monitoring efficacy [174, 175]. For example, evaluating the quantity and functional status of TILs can provide valuable insights for the application of PD1/PD-L1 inhibitors. Moreover, advancements in liquid biopsy technology enable the monitoring of patients’ molecular characteristics through blood samples, facilitating dynamic adjustments to treatment plans [176–178]. Long-term follow-up and management are also essential for personalized treatment. Regularly assessing patients’ treatment responses and adverse effects and promptly adjusting treatment strategies are crucial for achieving personalized treatment goals. In the future, follow-up and management tools based on big data and artificial intelligence may further enhance the precision and efficacy of personalized treatment [179]. The continued development of PD1/PD-L1 inhibitors in combination with personalized treatment strategies will help achieve precision medicine, extending patients’ survival and improving their quality of life.

Exploring novel therapies related to PD1/PD-L1 is also a current research hotspot. Monotherapy with ICIs does not meet the needs of all patients, and combination therapies may provide a breakthrough [180–182]. As previously discussed, research indicates that combining PD1 inhibitors with targeted therapies like TKIs can markedly enhance the prognosis of patients with ccRCC. Additionally, combining immunotherapy with radiotherapy has shown promising clinical outcomes. Strategies to modulate the tumor microenvironment, such as using CXCR4 inhibitors and antibody-drug conjugates (ADCs), are under clinical investigation and demonstrate potential therapeutic prospects [113]. Cutting-edge research has designed CAR-T cells capable of secreting anti-PD-L1 monoclonal antibodies (mAbs), known as immune-restorative (IR) CAR G36-PDL1, targeting carbonic anhydrase IX (CAIX). Analysis of the tumor microenvironment reveals that G36-PDL1 CAR-T cells enhance anti-tumor immunity by stimulating cytotoxic tumor-killing, decreasing immunosuppressive cells such as M2 macrophages and exhausted CD8 + T cells, and promoting interactions between Tfh cells and B cells. [183]. These novel therapeutic explorations not only expand the treatment options for ccRCC but also provide new avenues for future clinical practice.

In addition to novel therapies and precision treatments, an in-depth exploration of the mechanisms of PD1/PD-L1 in ccRCC is crucial, as it holds significant implications for uncovering resistance mechanisms and optimizing treatment strategies. Advanced methods, such as high-throughput sequencing, single-cell analysis, and in vivo and in vitro models, are being employed to elucidate the molecular mechanisms of the PD1/PD-L1 pathway in tumor immune evasion in ccRCC. The interaction between the PD1/PD-L1 pathway and other immune checkpoint molecules, as well as the impact of the tumor microenvironment on immunotherapy response, are key areas of current basic research. In-depth mechanistic studies can provide a theoretical basis for developing novel combination therapies and help identify biomarkers to predict therapeutic efficacy, ultimately enabling more precise treatments.

In summary, this review provides a comprehensive overview of the research on PD1/PD-L1 in ccRCC, covering aspects such as expression and functional regulation, clinical applications, and combination therapies. The PD1/PD-L1 pathway facilitates tumor immune evasion by suppressing T-cell function and is linked to unfavorable outcomes when highly activated. Clinical trials have demonstrated that PD1/PD-L1 inhibitors, like nivolumab, substantially enhance survival and response rates in advanced ccRCC patients, correlating closely with PD-L1 expression levels and tumor mutational burden. To overcome the issue of monotherapy resistance, researchers have explored the combination of PD1/PD-L1 inhibitors with other therapies, such as TKIs, CTLA-4 inhibitors, and emerging CXCR4 inhibitors, demonstrating higher efficacy and anti-tumor activity. Future research will further optimize these combination therapies, uncover the mechanisms of the PD1/PD-L1 pathway, and develop personalized treatment strategies based on patient characteristics to improve treatment outcomes and patient quality of life.

## Data Availability

No datasets were generated or analysed during the current study.

## Abbreviations

ccRCC: Clear cell renal cell carcinoma
VHL: Von Hippel-Lindau
APCs: Antigen-presenting cells
TILs: Tumor-infiltrating lymphocytes
TME: Tumor microenvironment
Tregs: Regulatory T cells
MDSCs: Myeloid-derived suppressor cells
TLS: Tertiary lymphoid structures
TINK: Tumor-infiltrating NK cells
TAMs: Tumor-associated macrophages
HIF2α: Hypoxia-inducible factor 2α
ICD: Immunogenic cell death
BSJPF: Bu Shen Jian Pi Fang
DFS: Disease-free survival
DSS: Disease-specific survival
OS: Overall survival
PFS: Progression-free survival
Tfh: T follicular helper cells
CPI: Checkpoint inhibitor
ORR: Overall response rate
SD: Stable disease
RTKIs: Receptor tyrosine kinase inhibitors
ACE2: Angiotensin-converting enzyme 2
EN: Endothelium-derived neurotrophic factor
CTL: Cytotoxic T lymphocyte
ICIs: Immune checkpoint inhibitors
ST-seq: Spatial transcriptome sequencing
NLR: Neutrophil-to-lymphocyte ratio
IES: Immune Exclusion Score
TMB: Tumor mutation burden
CCCP: Complement and coagulation cascades pathway
ITH: Intratumor heterogeneity
hERVs: Human endogenous retroviruses
TE: Transposable element
MSI: Microsatellite instability
ADCs: Antibody-drug conjugates
mAbs: Monoclonal antibodies
IR: Immune-restorative
CAIX: Carbonic anhydrase IX
